# Supplementary material for: Engineering subtilisin proteases that specifically degrade active RAS
Source: Commun Biol. 2021 Mar 5;4:299. doi: 10.1038/s42003-021-01818-7 (PMC7935941; doi:10.1038/s42003-021-01818-7)
Supplement: Supplementary file 2 — Supplementary Information [file 42003_2021_1818_MOESM2_ESM.pdf]

Supplemental Figures and Tables

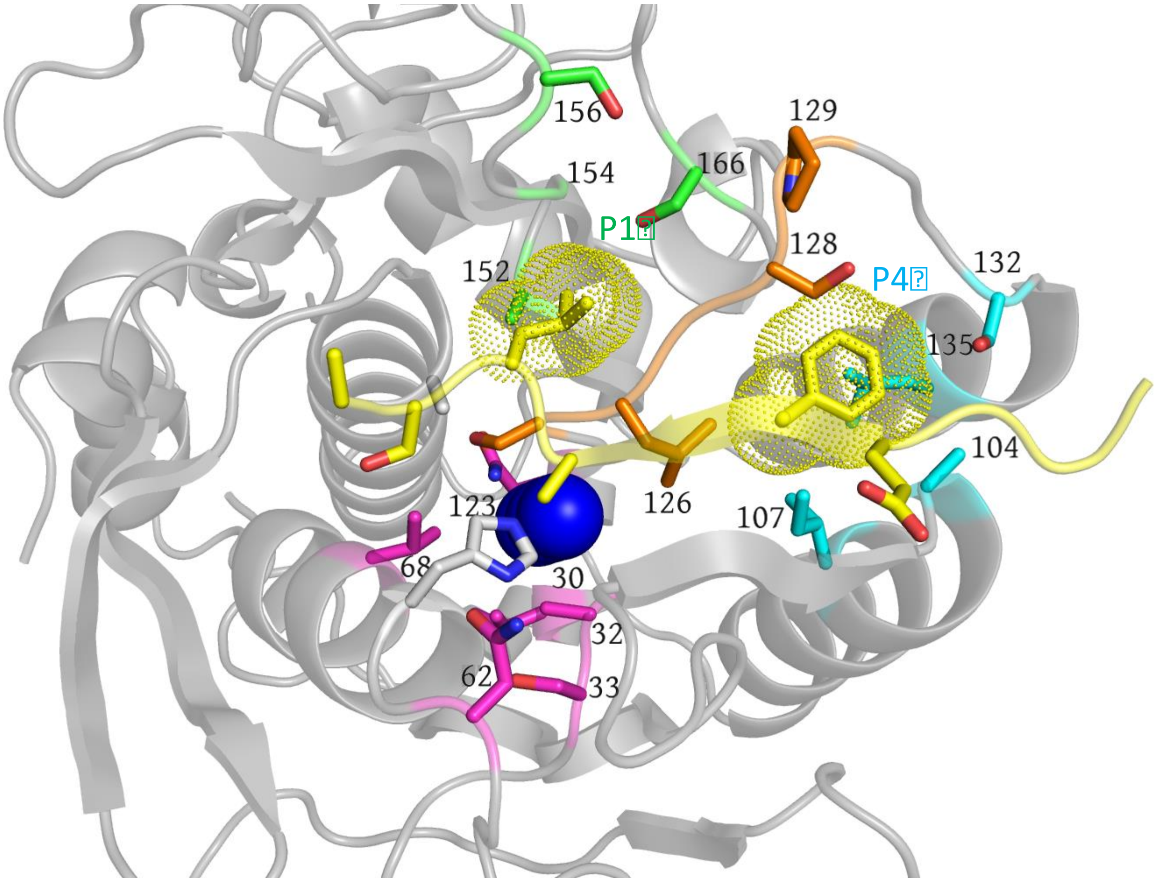

Fig. S1: *Sites modeled for mutagenesis* P1 pocket are in green, sites in the P4 pocket in cyan, and sites in the anion pocket are in violet. P1 leucine and P4 phenylalanine are shown with dot surfaces. The three binding sites are interconnected by common amino acids in the region from 123-129. These amino acids are in orange. Most subtilisin contacts are with the first five substrate amino acids on the acyl side of the scissile bond (denoted P1 through P5, numbering from the scissile bond toward the N-terminus of the substrate and the first amino acid on the leaving group side. Model based on 3BGO.pdb.

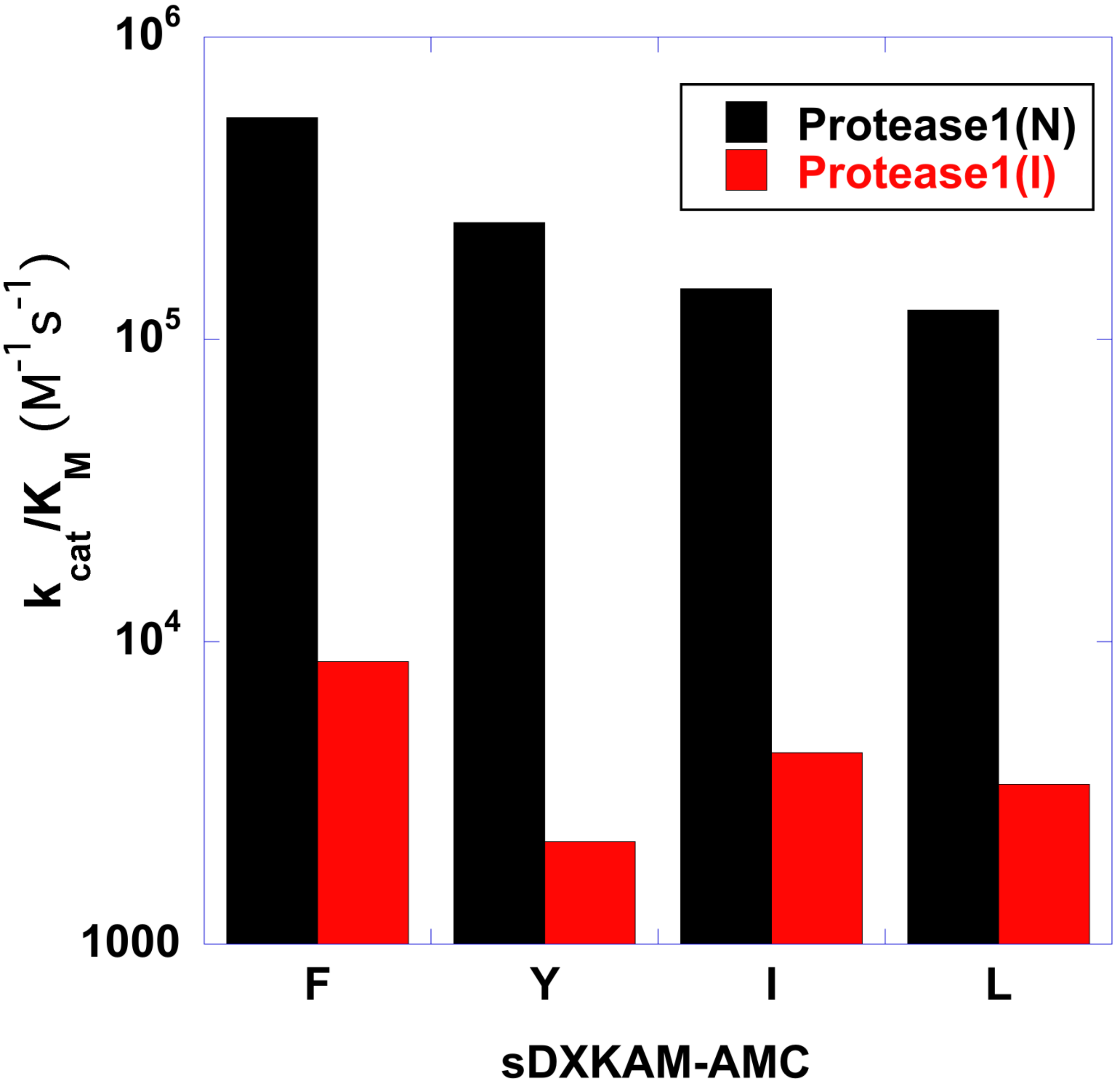

Fig. S2: *First generation proteases evaluated with DXKAM-AMC substrate series*. k_{cat}/K_M values are shown for hydrophobic amino acids at P4. Protease1(N): 10mM nitrite; Protease1(I): 10mM imidazole.

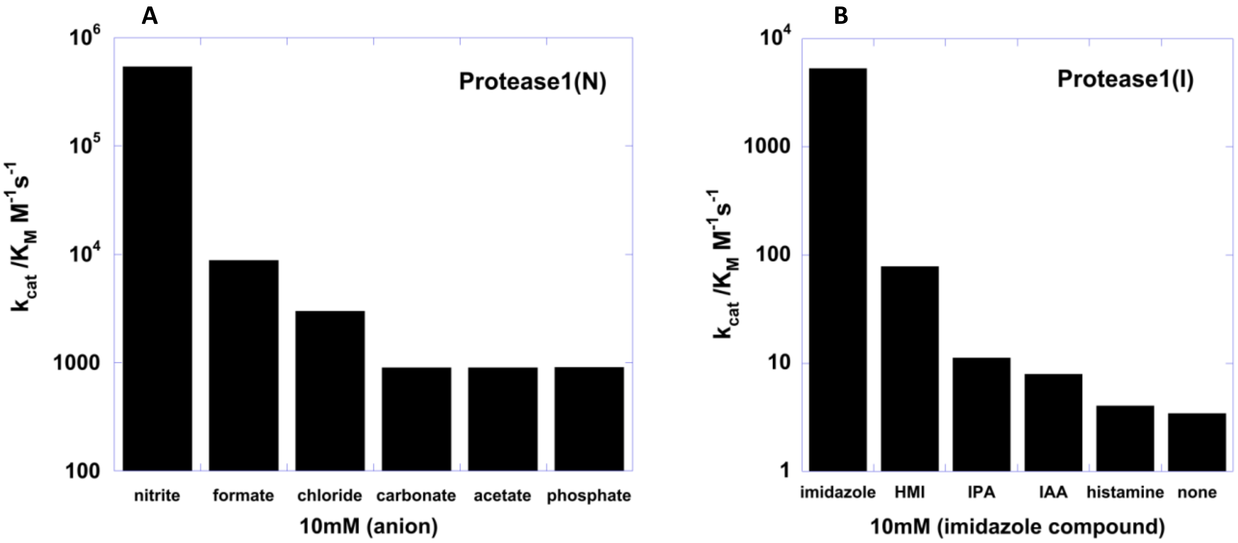

Fig. S3: *k_{cat}/K_M as a function of naturally-occurring anions and imidazole compounds for Protease1(N) and Protease1(I)*. HMI: 4-hydroxymethylimidazole (pKa = 6.45); IPA: imidazole-4-propionic acid (pKa = 6.77); IAA: imidazole-4-acetic acid (pKa = 6.78). The substrate was sDFKAM-AMC.

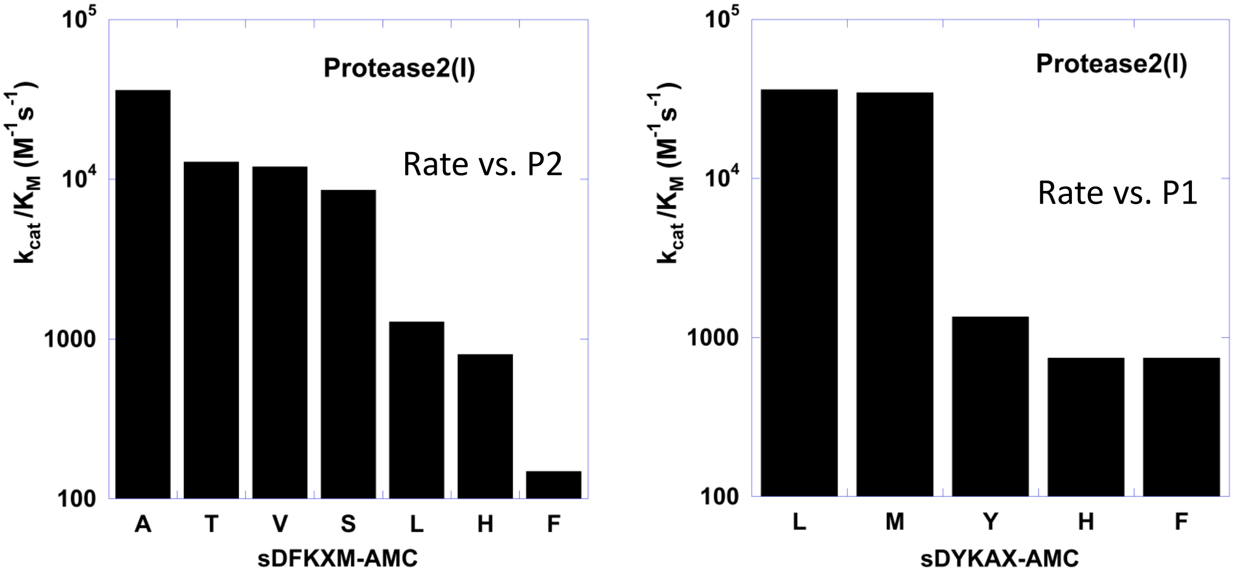

Fig. S4: *Evaluating specificities of P2 and P1 sub-sites.* Protease2(I) in 10mM imidazole evaluated with peptide-AMC substrates with variations at P2, and P1. Similar results were observed with Protease2(N) in 1mM nitrite.

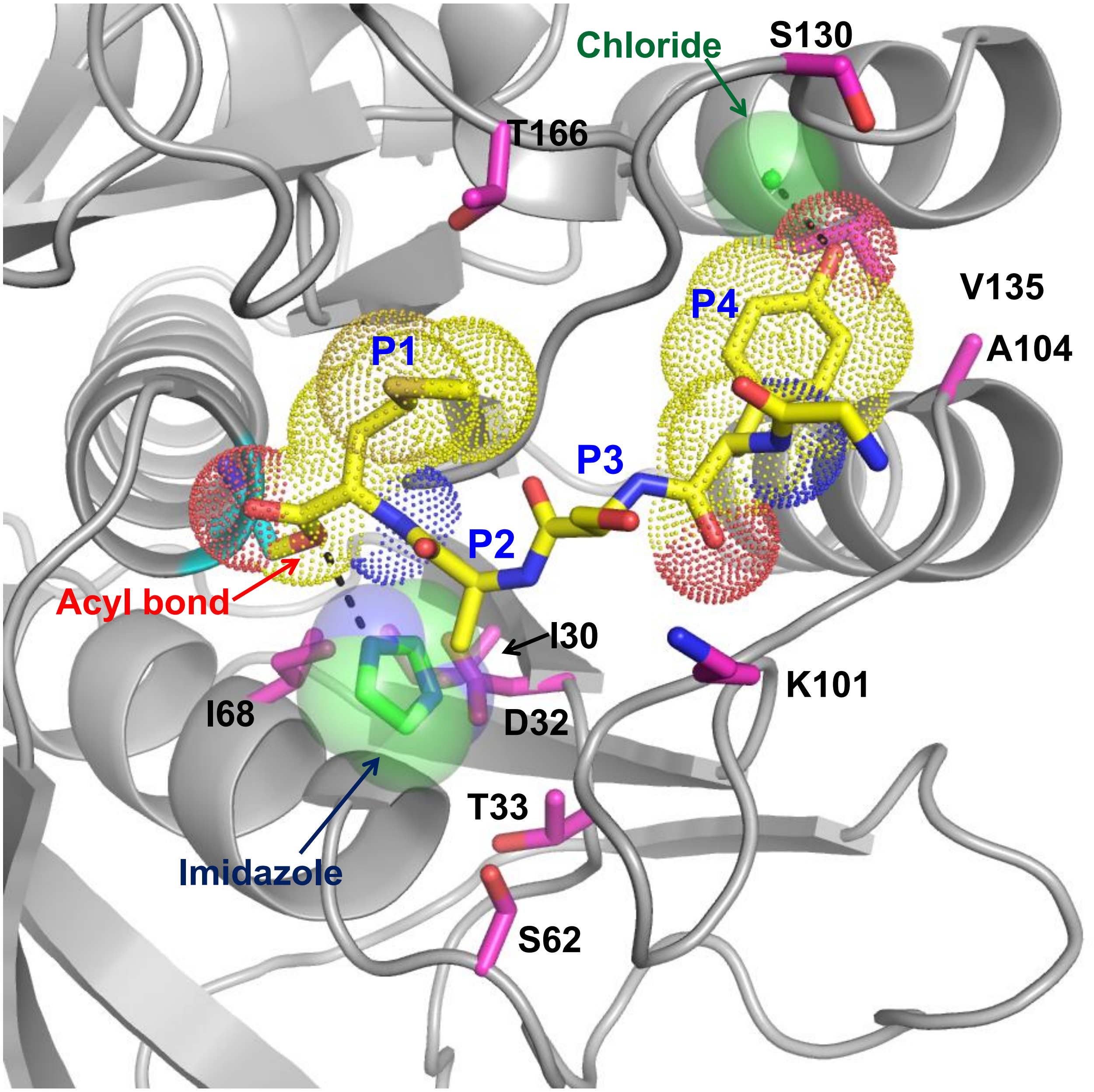

Fig S5: RASProtease(I) QEEYSAM with imidazole (green) modeled in place of three waters. New amino acids from the design process are in magenta. Substrate is in yellow. Only P1 to P4 are shown for clarity. An ion (modeled as chloride, pale green sphere) interacts with the hydroxyl groups of the P4 Tyr and Y171 of RASProtease(I).

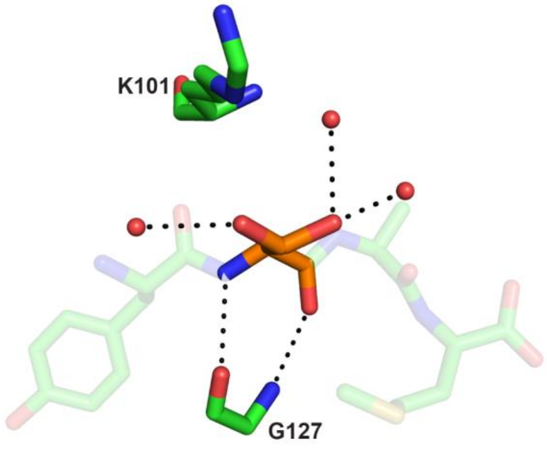

Fig. S6: *Changes to residue 101 affect substrate preference at P3 for RASProtease(I)* (left, P3=S) versus Protease1(N) (right, P3=R). Only P3 for the bound peptide is shown as solid sticks (orange) for clarity. The remaining residues are shown as semitransparent sticks.

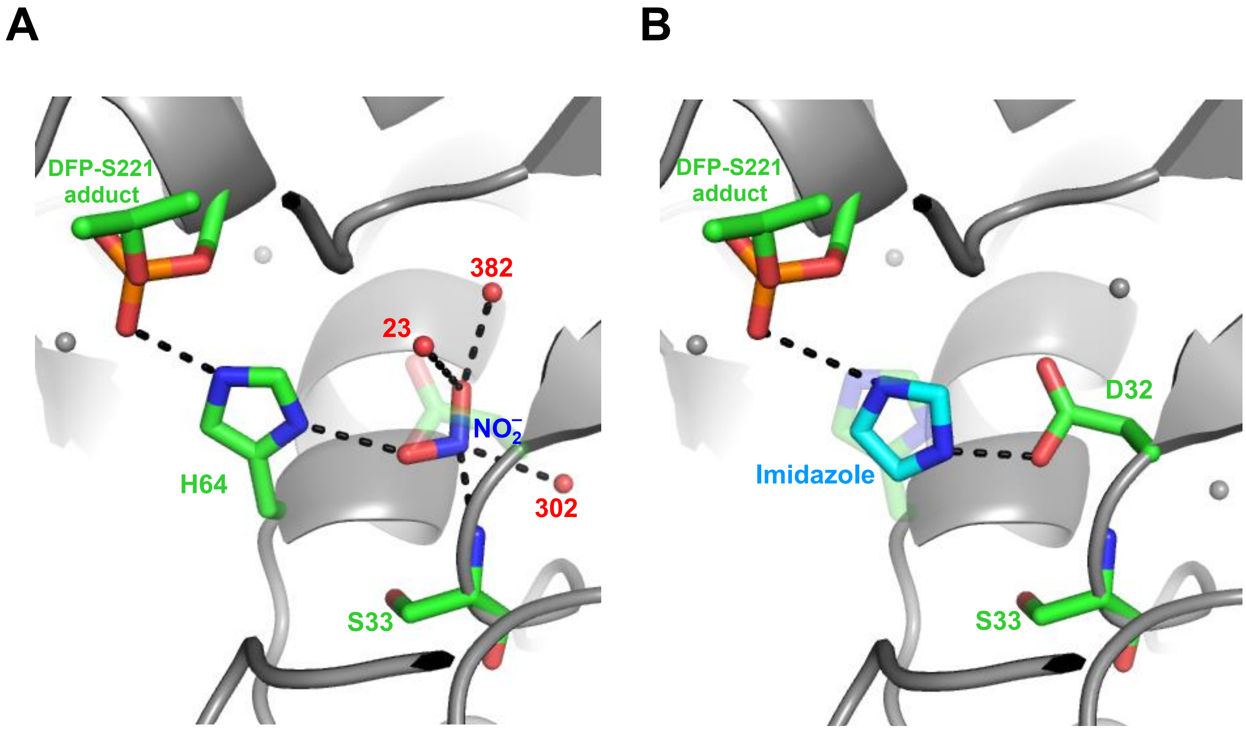

Fig. S7: *Catalytic triad with overlaid co-factor shows how cofactors substitute for the mutated catalytic residue* **A)** nitrite (modeled based on 1SUE.pdb), **B)** imidazole (from YRGLIM structure – Bryan, Orban, and Toth, unpublished). Water molecules coordinating to nitrite in A are shown as red spheres. DFP = diisopropyl fluorophosphate.

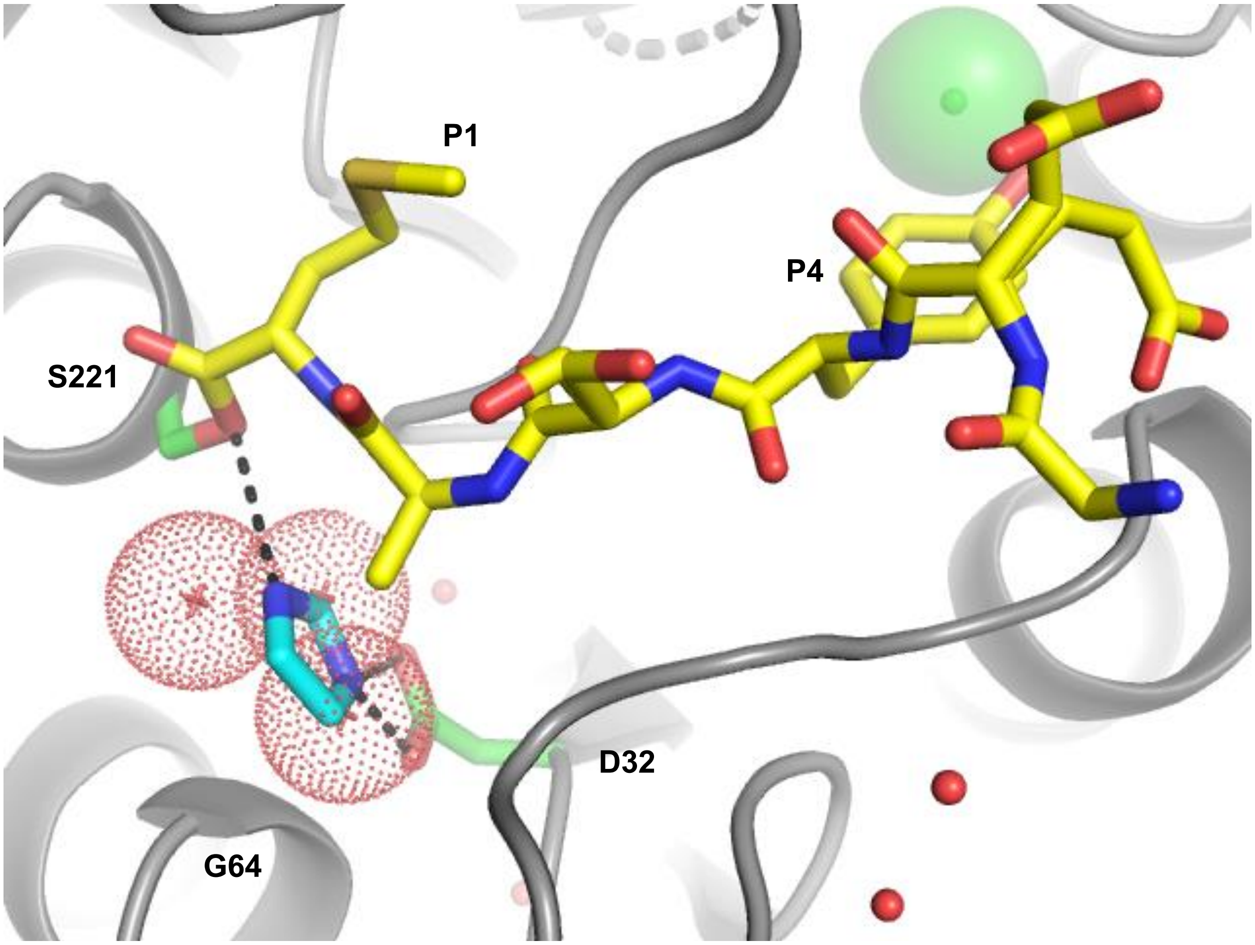

Fig S8: *QEEYSAM RASProtease(I) with imidazole in catalytic position*. Structures of RASProtease(I) without imidazole have three conserved waters (dot surfaces) that interact with Oδ1 and Oδ2 of D32, CO of S125, NH of G64, Oγ of S62, and Oγ of S221. When imidazole binds these waters are displaced, the imidazole nitrogens H-bond to Oδ1 and Oδ2 of D32 and Oγ of S221, and the charge relay system is reconstituted.

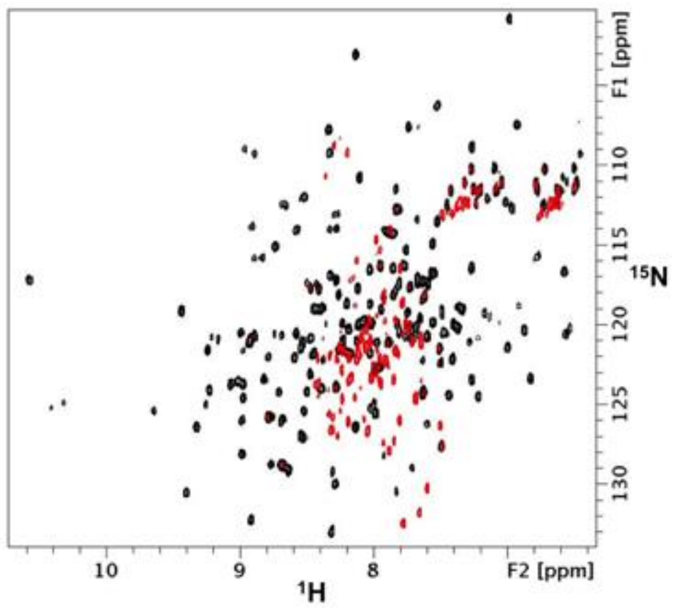

Fig. S9: *Overlaid 2D ^{1}H-^{15}N HSQC spectra of HRAS-G12V(GDP)* 100 μM concentration (black) and after treatment with 30 μM protease and 1 mM sodium nitrite at 37°C for 72 h (red). A control spectrum of the HRAS sample with no protease is unchanged under these conditions. The results indicate that cleavage at the YSAM site in the Switch 2 region of HRAS abolishes the globular fold, producing fragments with narrow ^{1}H shift dispersion that are consistent with disordered states.

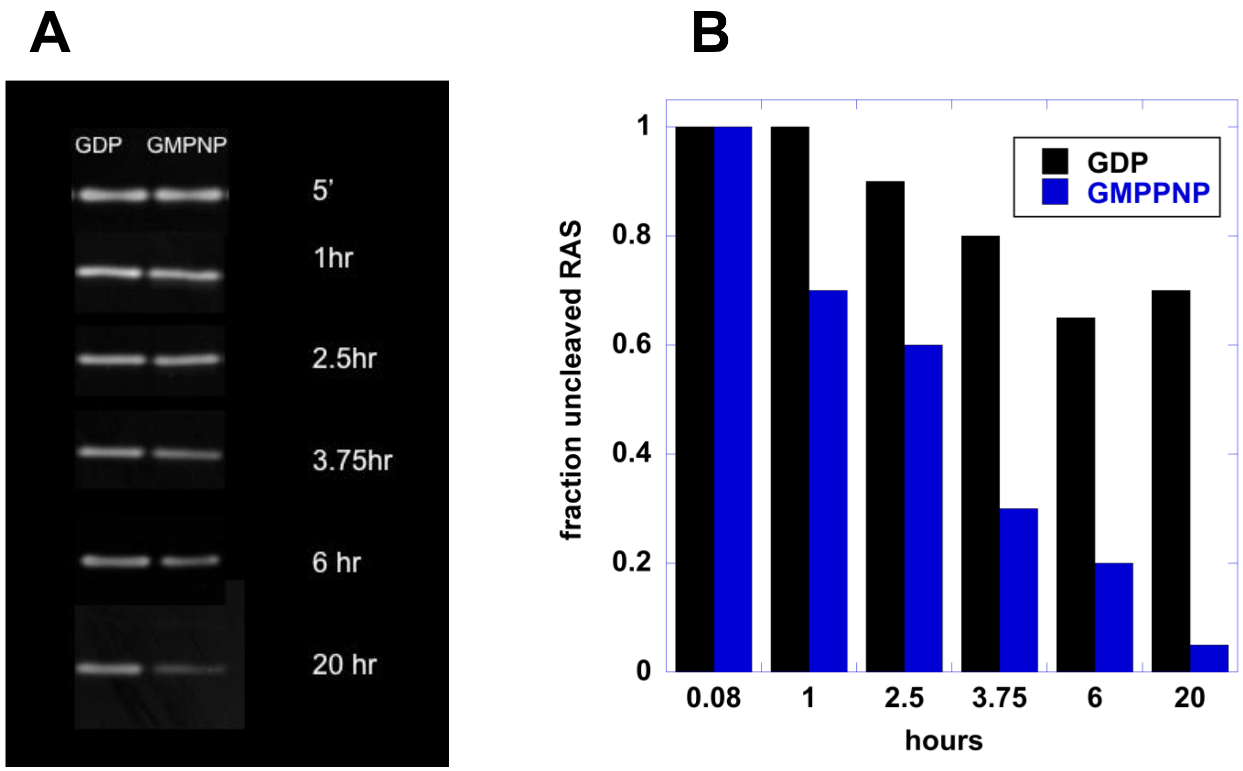

Fig. S10 : *Analysis of RAS cleavage* **(A)** The timing of RAS cleavage by 1 nM RASProtease(N) in 1 mM nitrite. At each time point (top to bottom), the 19,315 kDa RAS band from an SDS gel is shown for the RAS(GDP) and RAS(GMPNP) digests; (**B**) Normalized band density ± 10% (three replicates).

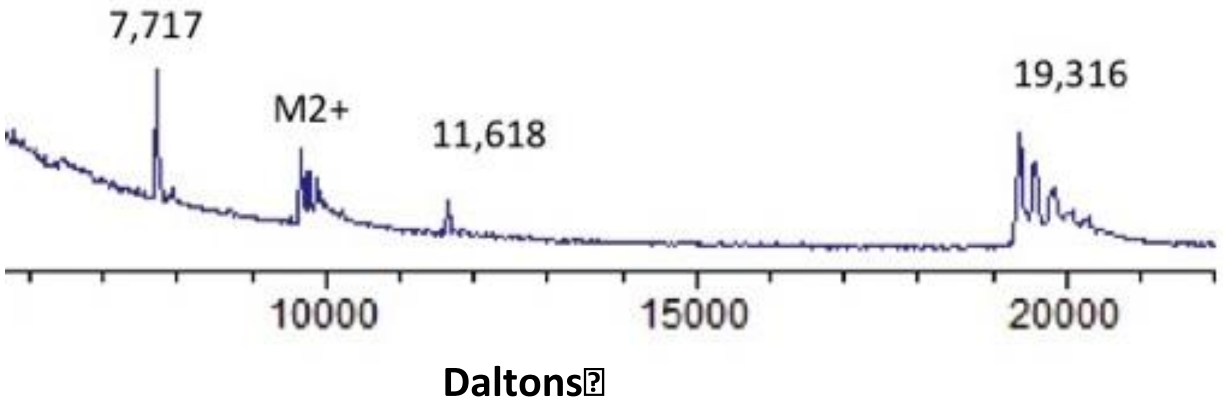

Fig. S11: *Analysis of HRAS cleavage.* MALDI analysis (20 hr time point of RAS(GDP) from Fig. S10. Intact HRAS is 19,315 Da, the N-terminal fragment is 7,716 Da, and the C-terminal fragment is 11,617 Da.

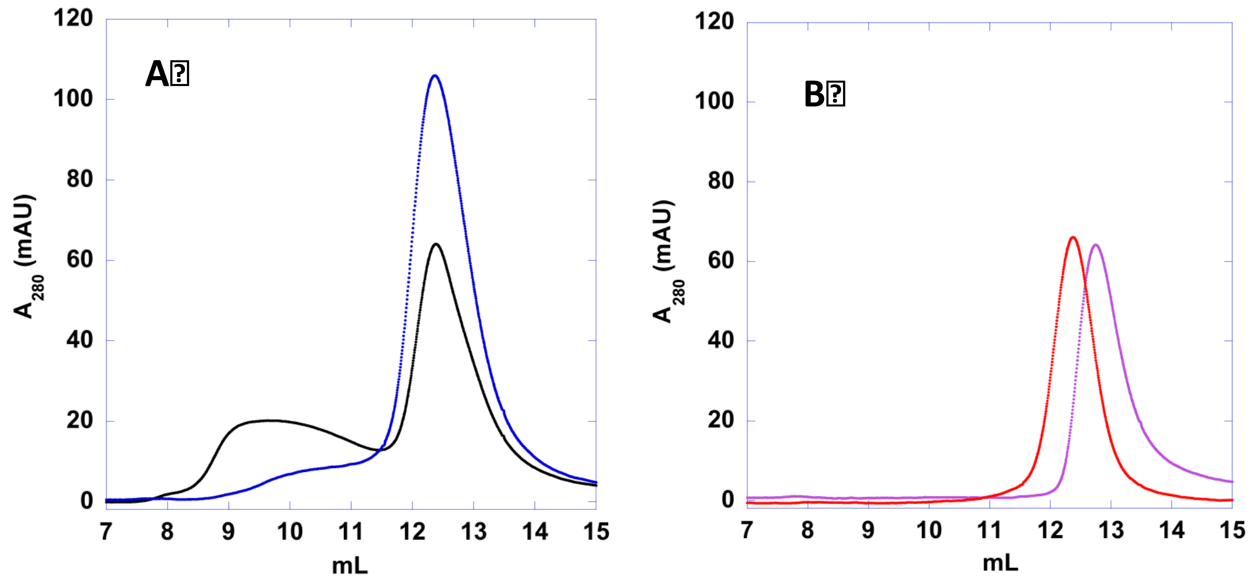

Fig. S12: *Gel filtration on G75* **A.** Stoichiometric mixtures of RASProtease(I) and RAS. 12.5µM RASProtease(I) - RAS(GDP) is in blue. 12.5µM RASProtease(I) - RAS(GMPPNP) is in black. **B.** Individual proteins. 12.5µM RASProtease(I) alone is in violet. 12.5µM RAS(GMPPNP) is in red. RAS(GDP) elutes identically to RAS(GMPPNP) and is not shown. Based on the fraction bound, K_S (without imidazole) is 6µM for RAS(GMPPNP) and 120µM for RAS(GDP). Molecular weight of RASProtease(I) = 26,438 daltons; RAS = 19,316 daltons (without cofactor).

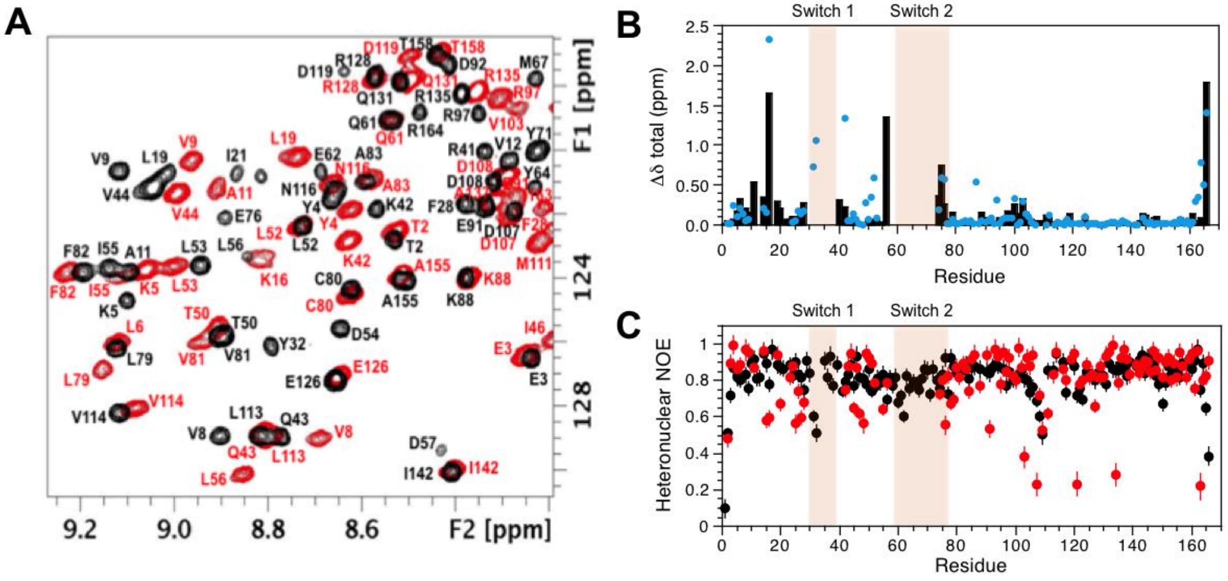

Fig. S13: *Structural and dynamic effects of guanosine nucleotide binding on HRAS.* (**A**) Overlaid 2D ^{1}H-^{15}N HSQC spectra showing downfield regions for the G12V variants of HRAS-GDP (black) and HRAS-GMPPNP (red). Backbone amide assignments are shown. F2, ^{1}H; F1, ^{15}N. Sample conditions are [HRAS] ~200 μM, 20 mM HEPES, 50 mM NaCl, 5 mM $MgCl_2$, 1 mM TCEP, pH 7.4, 298 K. (**B**) Changes in amide chemical shifts between G12V-HRAS-GDP and G12V-HRAS-GMPPNP (black). Shift perturbations between WT-KRAS-GDP and WT-KRAS-GMPPNP are also shown for comparison (blue) (41, 43). (**C**) {^{1}H}-^{15}N steady state heteronuclear NOE values for G12V-HRAS-GDP (black) and G12V-HRAS-GMPPNP (red).

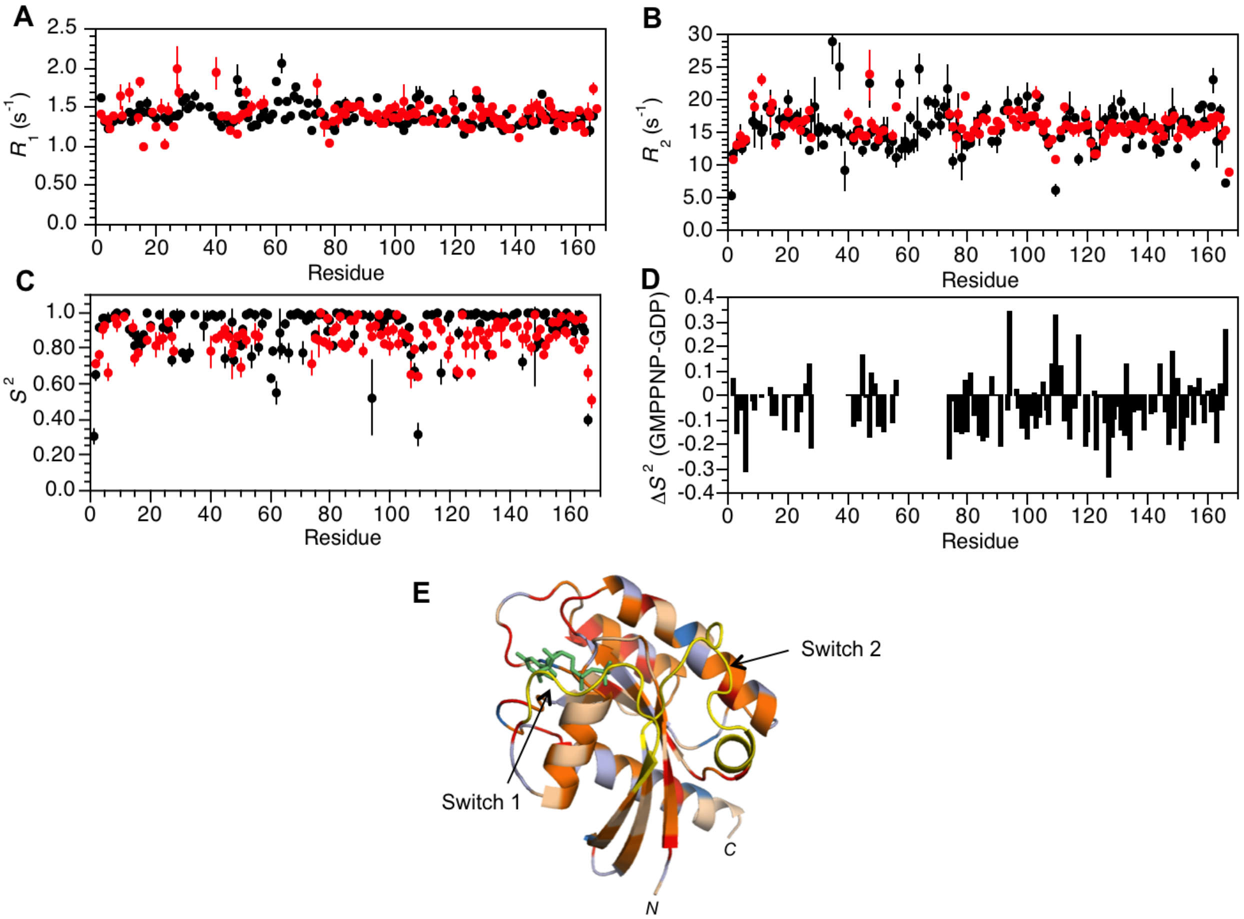

Fig. S14: *Changes in HRAS backbone dynamics as a function of nucleotide type.* (**A**) ^{15}N-Longitudinal (R_1) relaxation rates for G12V-HRAS versus residue number. Color-coding for (A-C) is G12V-HRAS-GDP (black) and G12V-HRAS-GMPPNP (red). Error bars indicate ± 1SD. (**B**) ^{15}N-Transverse (R_2) relaxation rates. (**C**) Order parameters (S^2) obtained from the ^{15}N relaxation data, including steady state heteronuclear NOE data in **Fig. S11**, using a model-free formalism. (**D**) Differences in order parameters (ΔS^2 = G12V-HRAS-GMPPNP – G12V-HRAS-GDP) versus residue number. Negative values indicate increased main chain flexibility while positive values indicate decreased flexibility in the GMPPNP-bound form. (**E**) ΔS^2 values mapped onto the structure of HRAS (PDB 2Q21). Color coding is as follows: $0>\Delta S^2>-0.15$, orange; $\Delta S^2<-0.15$, red; $0<\Delta S^2<0.15$, light blue; $\Delta S^2>0.15$, blue. The Switch 1 and Switch 2 regions, which are exchanged broadened in G12V-HRAS-GMPPNP, are highlighted in yellow. The nucleotide is shown in green.

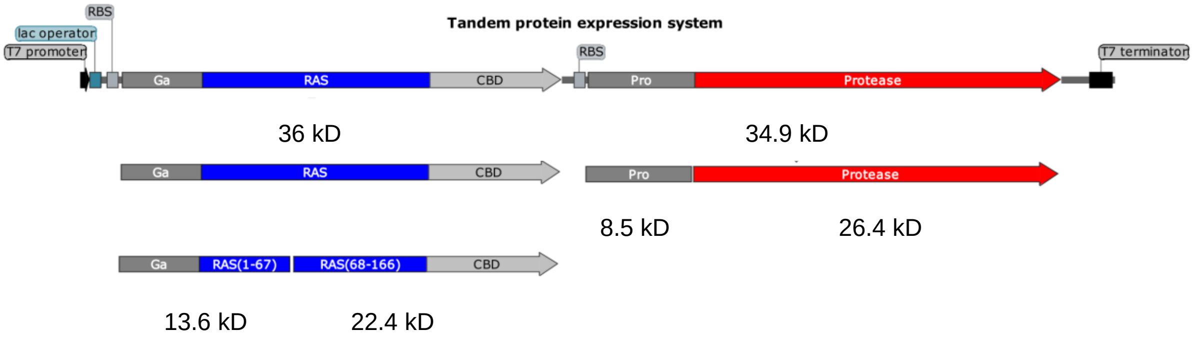

Fig. S15: *Gene for HRAS fusion protein co-expression with protease zymogen (pro-protease)*. The intact WT fusion protein is 35,974 daltons. The N- and C- terminal fragments are 13,579 daltons and 22,413 daltons, respectively.

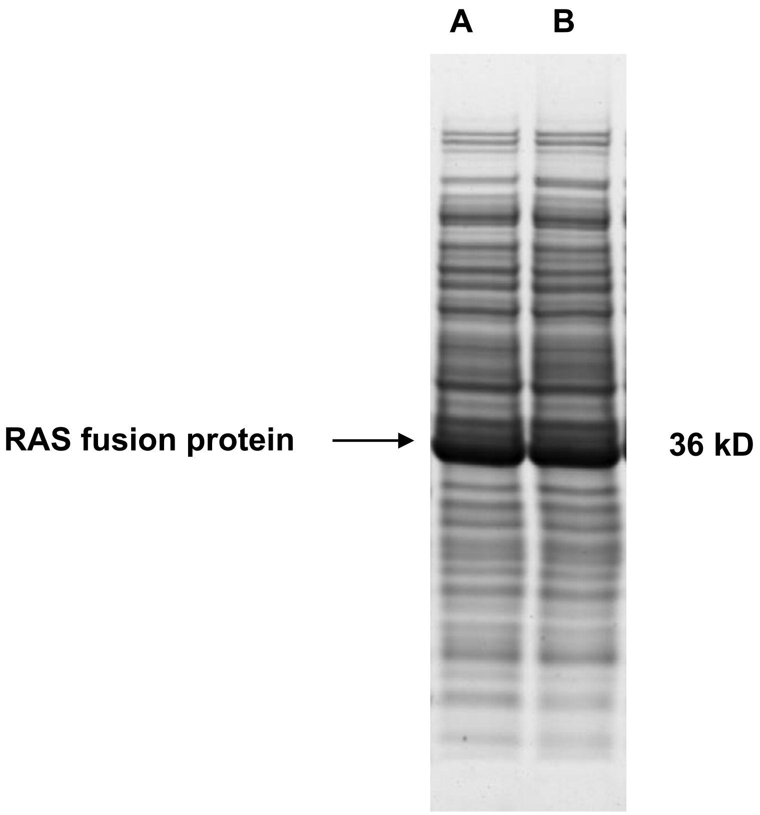

Fig. S16: *E. coli expression of RAS* Lanes A-B: RAS at 24hrs (**A**) and 42hrs (**B**).

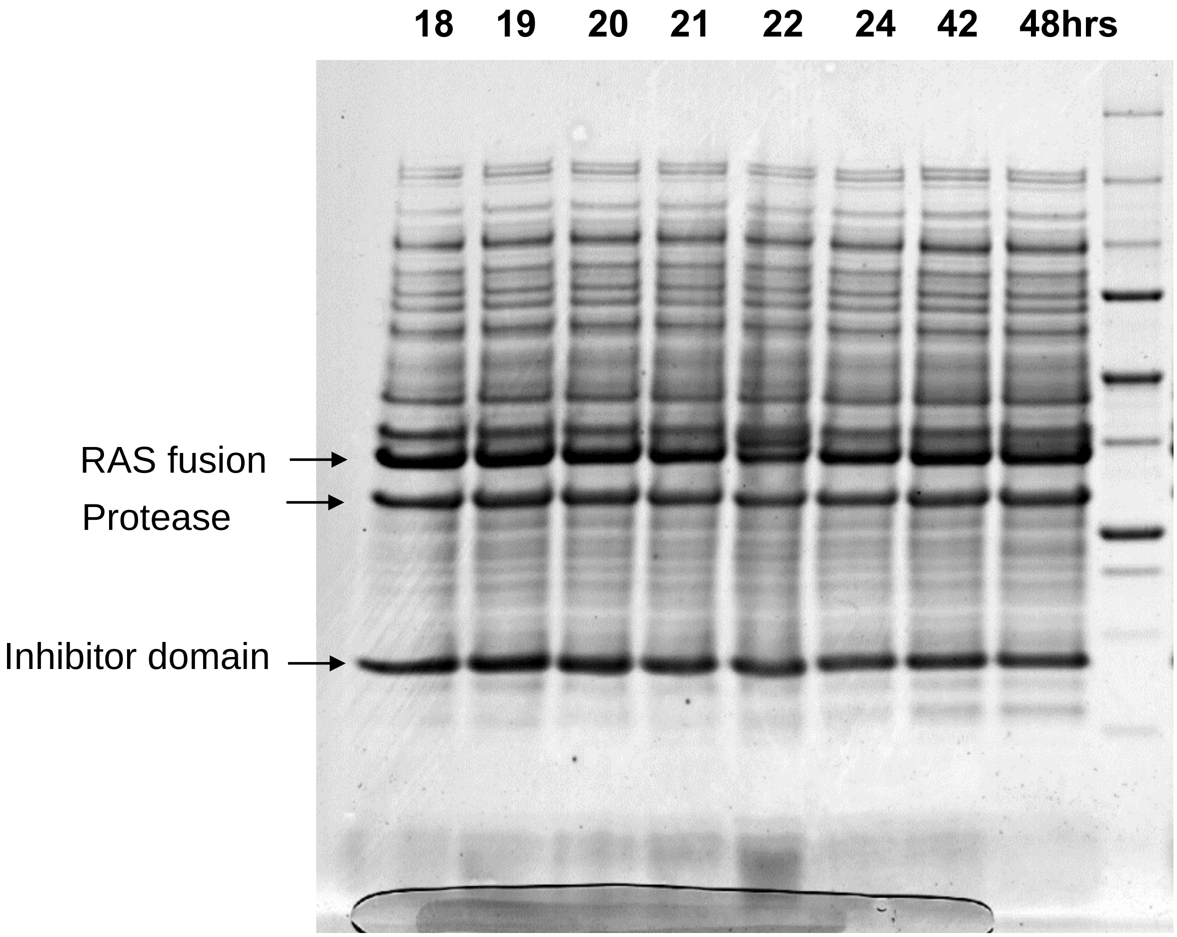

Fig. S17*:* ***RASProtease(I)*** *co-expressed with RAS without imidazole.* Culture times are as indicated. Intact RAS fusion protein is 35,974 daltons. Markers: 250, 150, 100, 75, 50, 37, 25, 20, 15, 10 kDa.

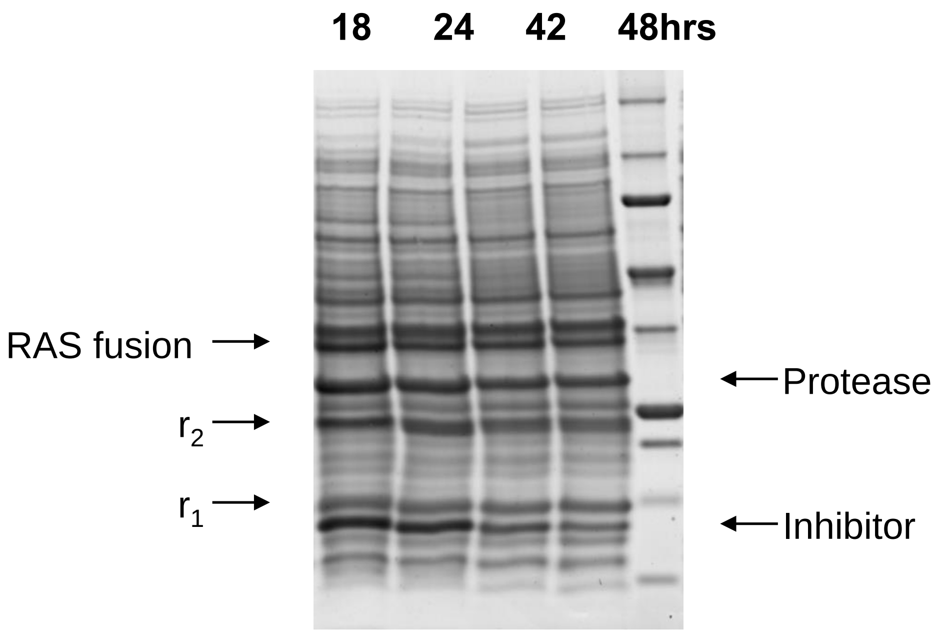

Fig. S18: *RASProtease(N) zymogen co-expressed with RAS and endogenous nitrite*. Culture times are as indicated. Intact RAS fusion protein is 35,974 daltons. The N- and C- terminal fragments are 13,579 daltons (r1) and 22,413 daltons (r2), respectively.

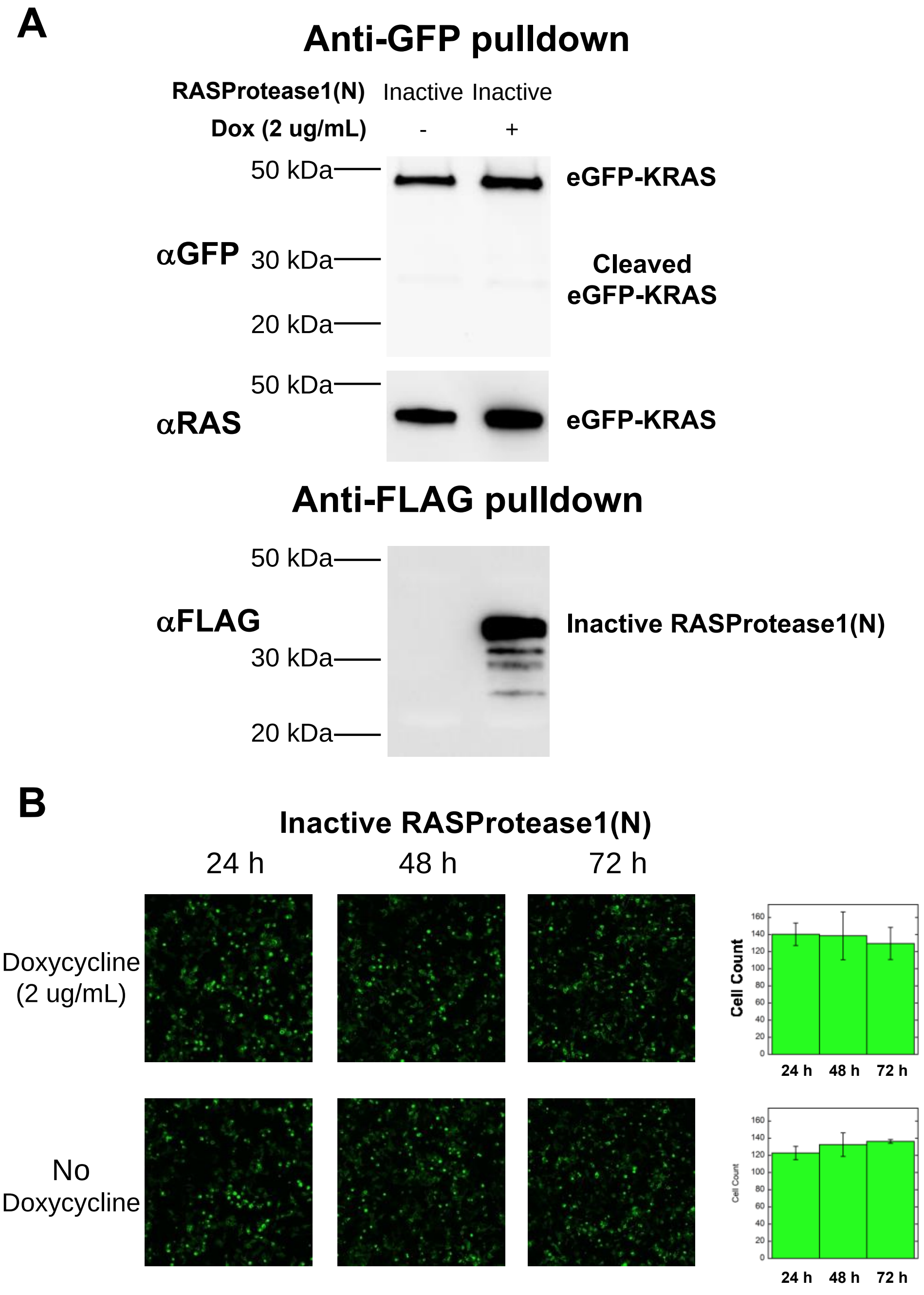

Fig. S19: *RAS-specific protease activity in cells* (**A**) Western blot analysis of cells co-transfected with eGFP-KRAS and inactive (S221A RASProtease does not result in the appearance of a KRAS cleavage product upon induction of the inactive protease when probed with an anti-GFP antibody following a GFP pull-down. Likewise, the intensity of the RAS-reactive band remains constant when probed with an anti-RAS antibody. The inactive protease fails to cleave its inhibitory I domain as evidenced by the unprocessed protease band at approximately 35 kDa. (**B**) Induction of the inactive protease in HEK 293T cells at 24 hours after transfection results in no change in GFP fluorescence at 48 and 72 hours after transfection compared to the same cells without induction of protease expression.

Table S1: Engineering progression and mutations in the catalytic, S1, and S4 regions

Protease*/position	30	32	33	62	64	68	101	104	107	128	135	166
subtilisin BPN’	V	D	S	N	H	V	S	Y	I	G	L	G
SBT189	V	A	S	N	H	V	S	A	I	S	L	S
Protease1(N)	I	G	T	S	H	I	S	A	V	S	L	T
Protease1(I)	I	D	T	S	G	I	S	A	V	S	L	T
Protease2(N)	I	G	T	S	H	I	S	A	I	S	V	T
Protease2(I)	I	D	T	S	G	I	S	A	I	S	V	T
RASProtease(N)	I	G	T	S	H	I	K	A	I	S	V	T
RASProtease(I)	I	D	T	S	G	I	K	A	I	S	V	T

*Engineered proteases have mutations at 18 positions in addition to those specified above [17].

Table S2: Kinetic parameters for RAS(GMPPNP) and RAS(GDP)

Protease	cofactor	k_2 /K_S ($M^{-1}s^{-1}$)	K_P (nM)
RASProtease(I)/RAS(GDP)	1mM imidazole	220	30
RASProtease(I)/RAS(GMPPNP)	1mM imidazole	13,720	30
RASProtease(N)/RAS(GDP)	1mM nitrite	410	40
RASProtease(N)/RAS(GMPPNP)	1mM nitrite	32,430	40

K_P is ± 5%.
