# Supplementary material for: Engineering subtilisin proteases that specifically degrade active RAS
Source: Commun Biol. 2021 Mar 5;4:299. doi: 10.1038/s42003-021-01818-7 (PMC7935941; doi:10.1038/s42003-021-01818-7)
Supplement: Supplementary file 3 — Description of Additional Supplementary Files [file 42003_2021_1818_MOESM3_ESM.pdf]

Description of Additional Supplementary Items

File Name: Supplementary Data 1

Description: Source data for the main figures
